# Supplementary material for: Systematic mapping of protein‐metabolite interactions in central metabolism of Escherichia coli
Source: Mol Syst Biol. 2019 Aug 23;15(8):e9008. doi: 10.15252/msb.20199008 (PMC6706640; doi:10.15252/msb.20199008)
Supplement: Supplementary file 1 — Appendix [file MSB-15-e9008-s001.pdf]

# Appendix

*Systematic mapping of protein-metabolite interactions in central metabolism of Escherichia coli.*
Maren Diether, Yaroslav Nikolaev, Frédéric H.T. Allain, Uwe Sauer

## Table of Contents


| Item | Title | Page |
|---|---|---|
| Appendix Figure S1 | Reference spectra for metabolite mix 1. | 2 |
| Appendix Figure S2 | Reference spectra for metabolite mix 2. | 3 |
| Appendix Figure S3 | Reference spectra for metabolite mix 3. | 4 |
| Appendix Figure S4 | Reference spectra for metabolite mix 4. | 5 |
| Appendix Figure S5 | Receiver-operator characteristics curve for the recovery of known interactions. | 6 |
| Appendix Figure S6 | Number of detected interactions per enzyme. | 7 |
| Appendix Figure S7 | Number of detected interactions per enzyme versus native protein size. | 8 |
| Appendix Figure S8 | Correlation of protein and metabolite hydrophobicity with number of detected interactions. | 9 |
| Appendix Figure S9 | Chemical similarity of new and known interactions. | 10 |
| Appendix Figure S10 | Comparison between *in vivo* and *in vitro* metabolite concentrations. | 11 |
| Appendix Figure S11 | Correlation of interactions detected per enzyme and metabolite in this study and Piazza et al. 2018. | 12 |
| Appendix Figure S12 | SDS-PAGE of all tested proteins. | 13 |
| Appendix Figure S13 | Raw data for enzyme assays with Pta and FbaA. | 14 |

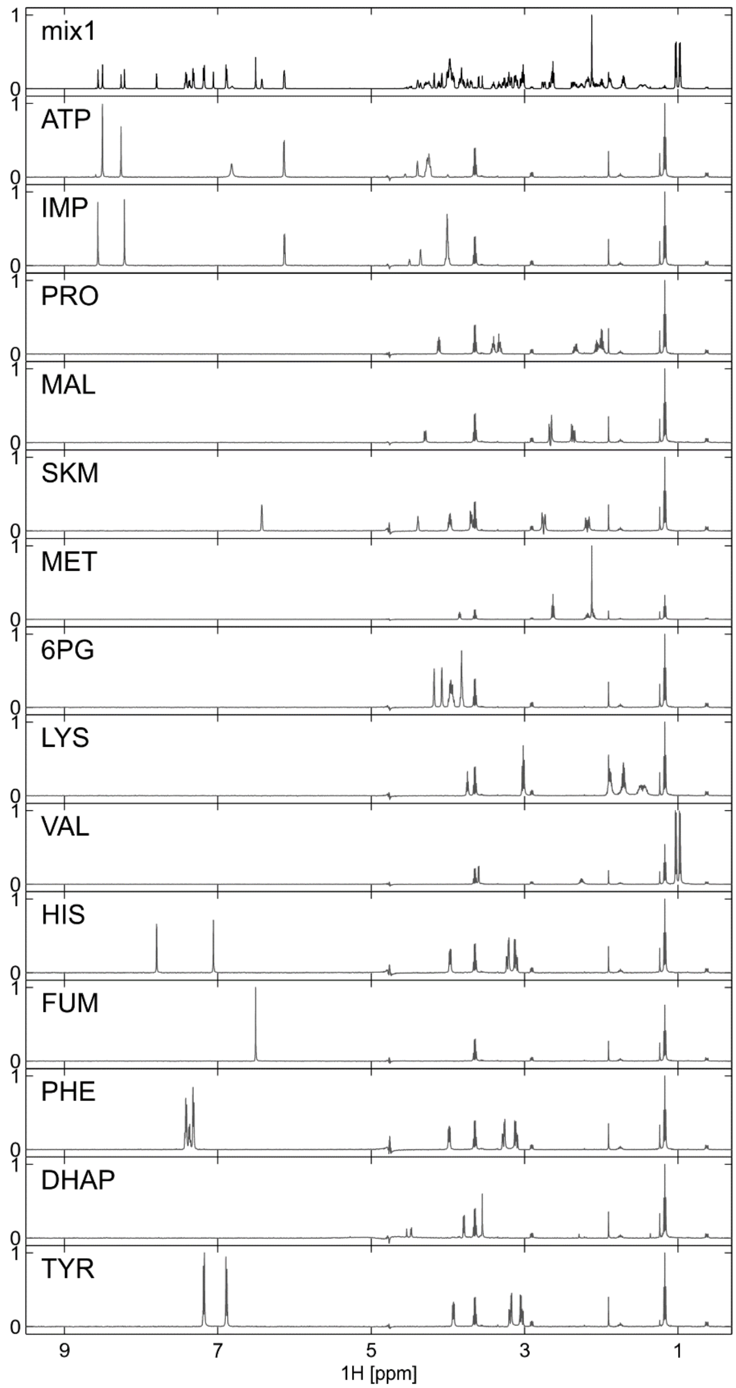


**Appendix Figure S1: Reference spectra for metabolite mix 1.**

1D $^{1}$H-NMR spectra of metabolite mix 1 and the individual metabolites contained therein.

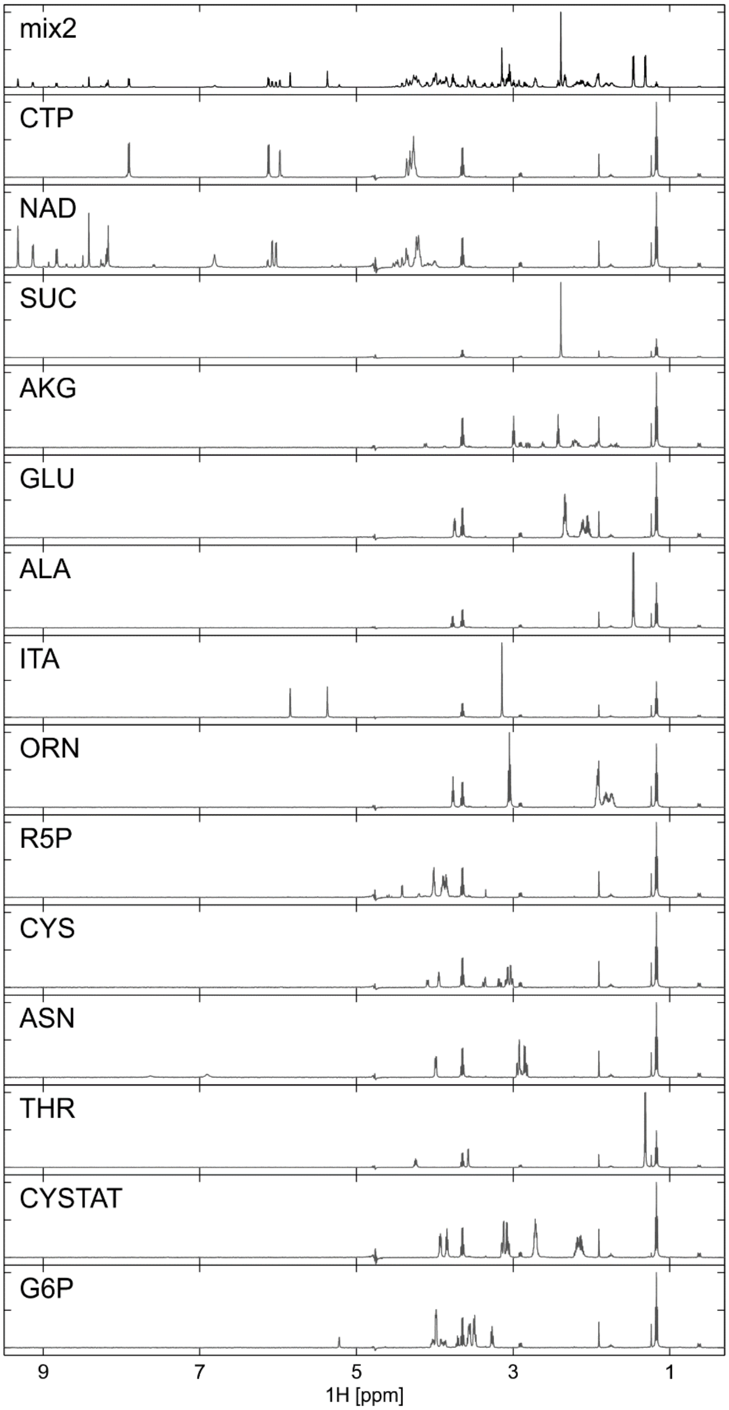


**Appendix Figure S2: Reference spectra for metabolite mix 2.**

1D $^1$H-NMR spectra of metabolite mix 2 and the individual metabolites contained therein.

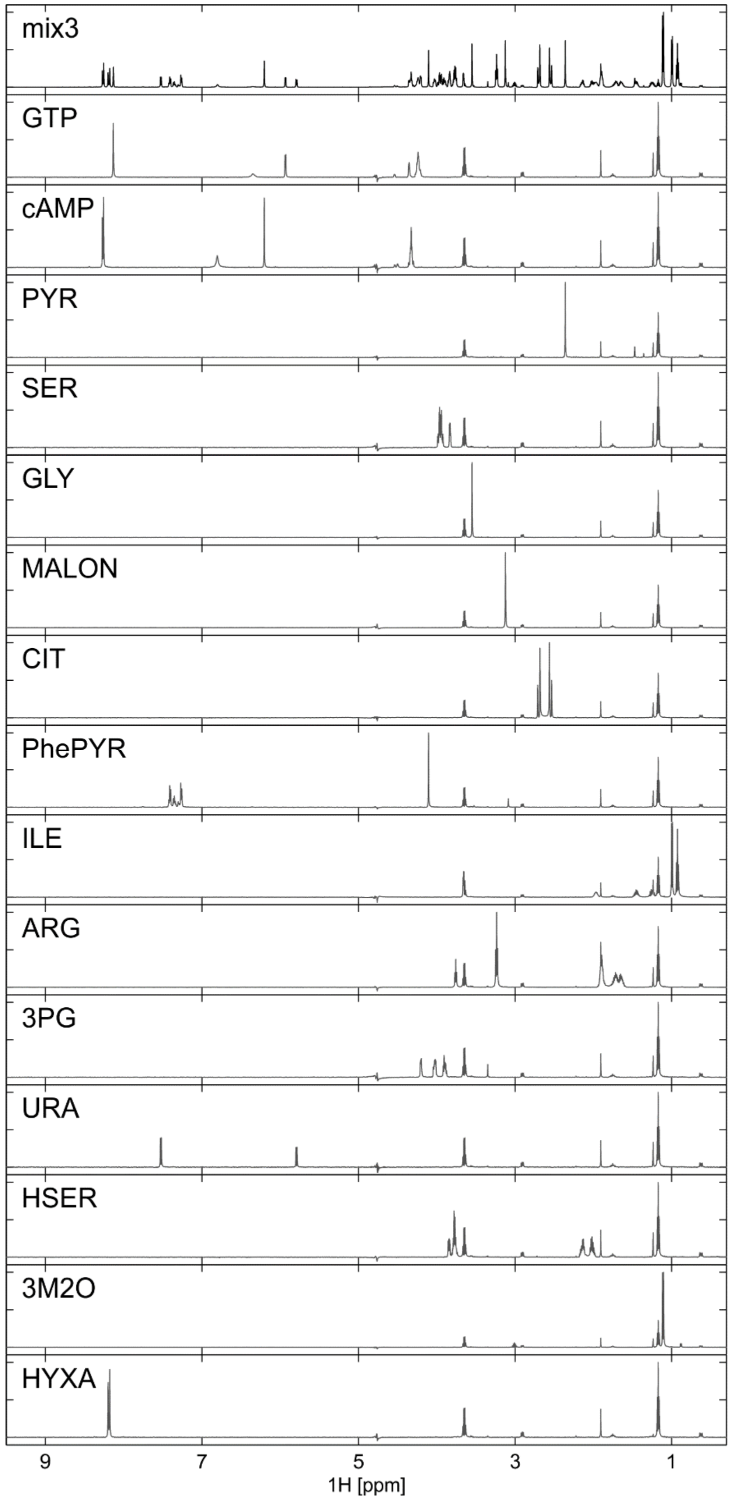


**Appendix Figure S3: Reference spectra for metabolite mix 3.**

1D $^{1}$H-NMR spectra of metabolite mix 3 and the individual metabolites contained therein.

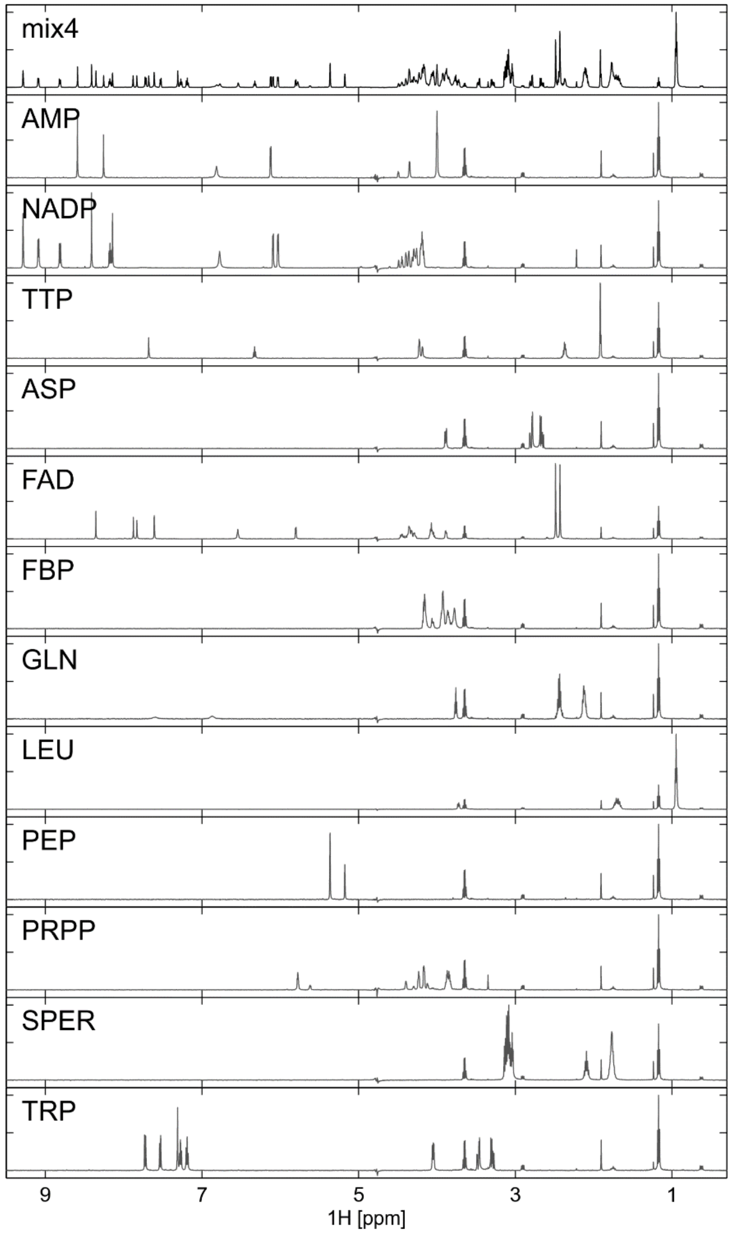


**Appendix Figure S1: Reference spectra for metabolite mix 4.**

1D $^{1}$H-NMR spectra of metabolite mix 4 and the individual metabolites contained therein.

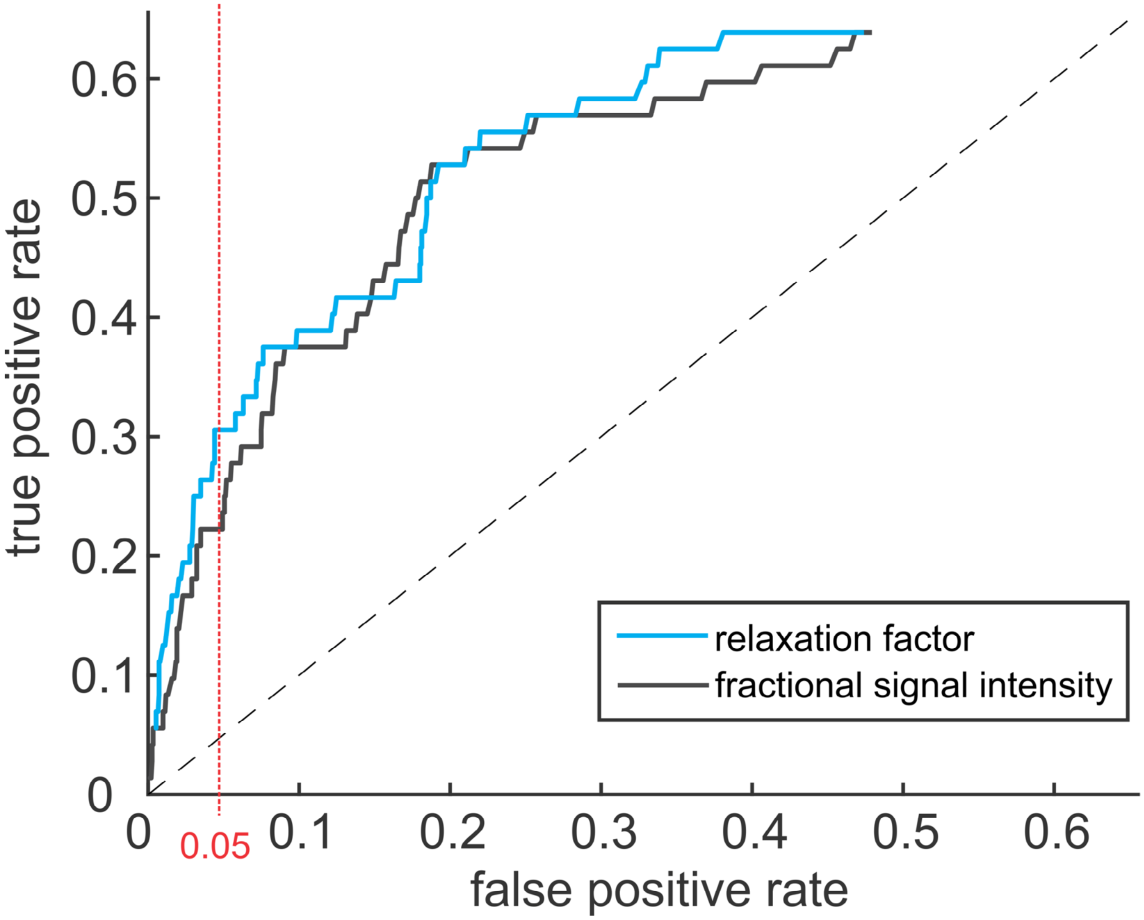


**Appendix Figure S5: Receiver-operator characteristics curve for the recovery of known interactions.**

The false-positive and true-positive rates of recovering known interactions from EcoCyc were calculated for different cutoffs of the relaxation factor ΔRF (blue line) and the fractional signal intensity (black line).

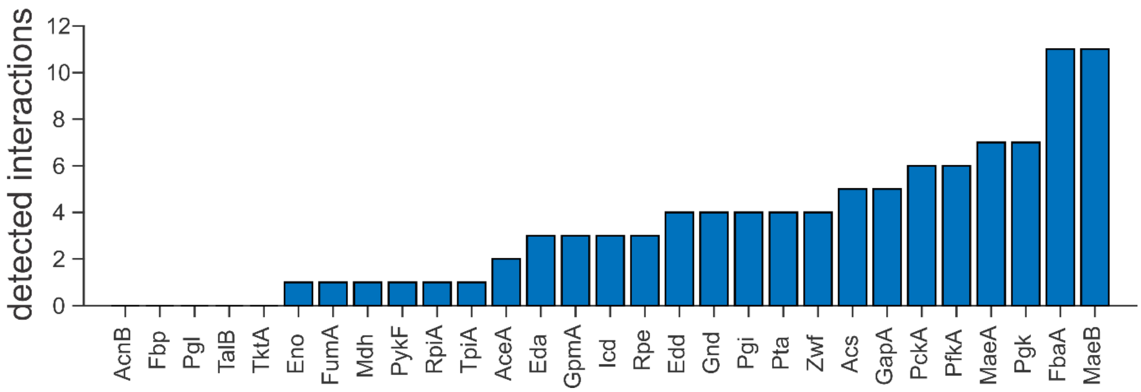


**Appendix Figure S6: Number of detected interactions per enzyme.**

The height of the bars indicate the number of interactions detected with NMR.

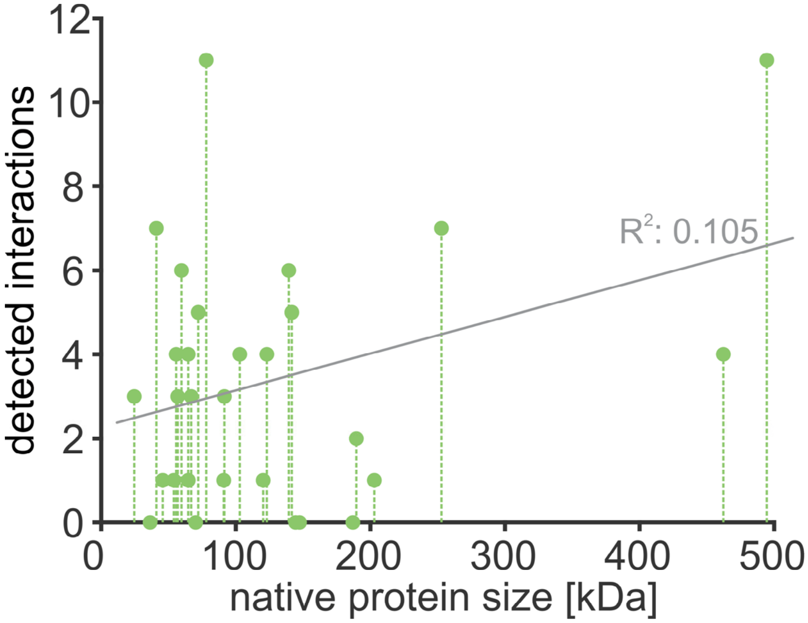


**Appendix Figure S7: Number of detected interactions per enzyme versus native protein size.**

The detected interactions per protein (ΔRF > 0.1805) are plotted against the native protein size. The grey line represents a linear fit with $R^2 = 0.105$.

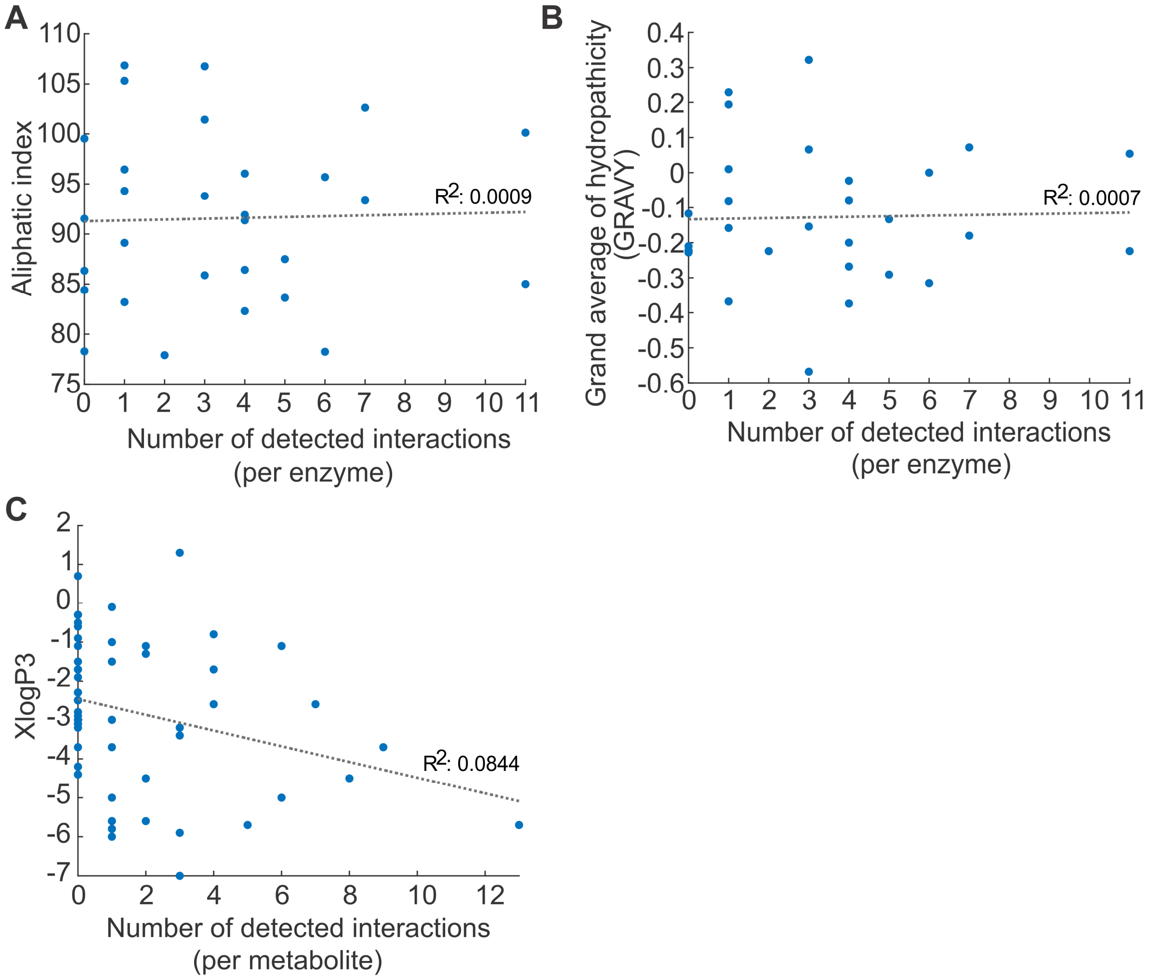


**Appendix Figure S8: Correlation of protein and metabolite hydrophobicity with number of detected interactions.**

**A**, Correlation of protein aliphatic index (from sequence level analysis) with the number of detected interactions per protein. The grey dotted line represents a linear fit with $R^2$ = 0.0009.

**B**, Correlation of protein grand average of hydropathicity (from sequence level analysis) with the number of detected interactions per protein. The grey dotted line represents a linear fit with $R^2$ = 0.0007.

**C**, Correlation of metabolite lipophilicity (XLogP3, from PubChem) with the number of detected interactions per metabolite. The grey dotted line represents a linear fit with $R^2$ = 0.0844.

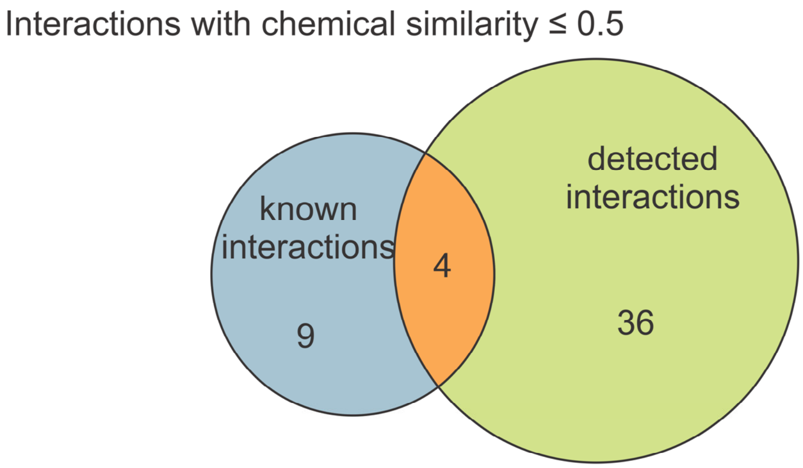


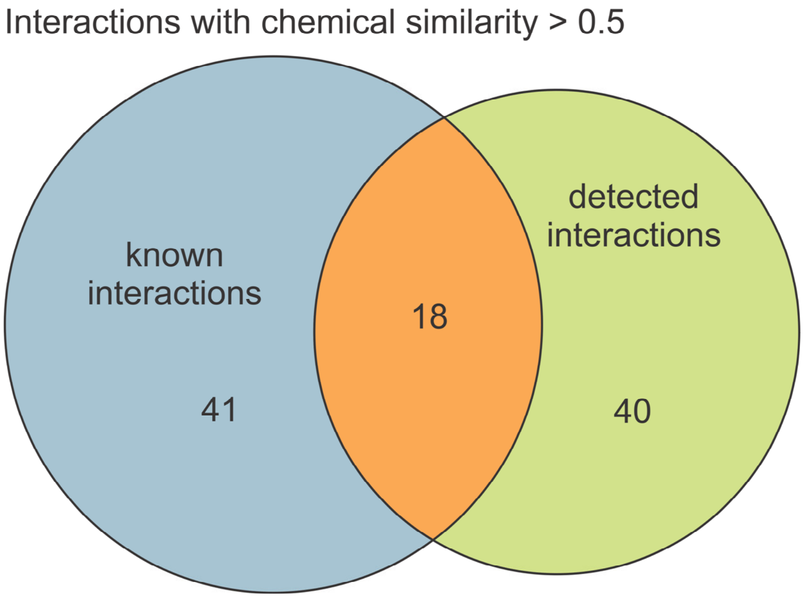


**Appendix Figure S9: Chemical similarity of new and known interactions.**

For metabolite enzyme-interactions with a score above 0.5 and below 0.5 the number of known, known and detected and newly detected interactions is presented in blue, orange and green respectively.

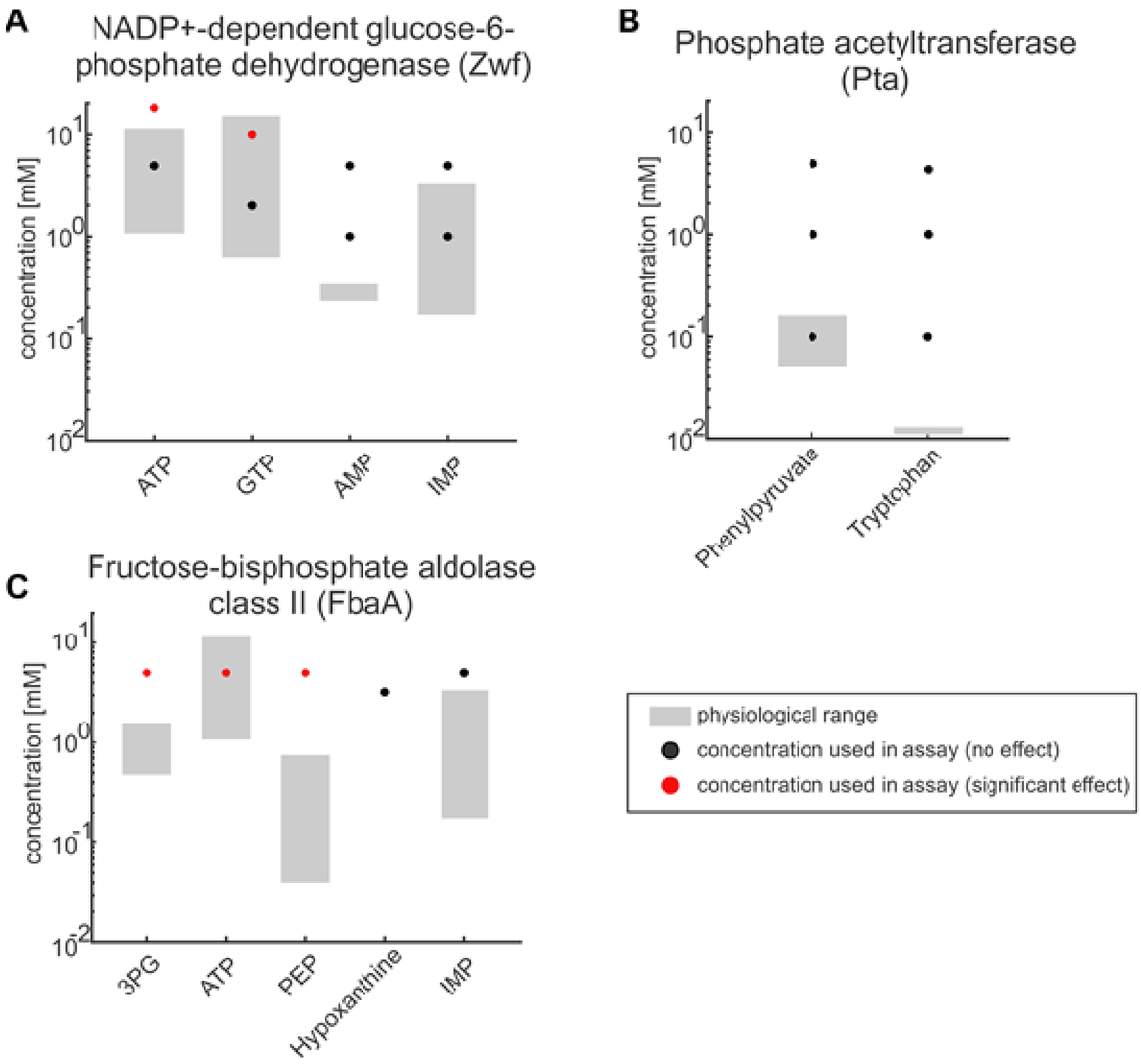


**Appendix Figure S10: Comparison between *in vivo* and *in vitro* metabolite concentrations.**

The grey bar denotes the *in vivo* steady state concentrations of all metabolites used in *in vitro* enzyme assays. Data was extracted from Park et al, 2016 and Kochanowski et al, 2017, for hypoxanthine, no *in vivo* concentration was available.

A, *In vivo* and *in vitro* metabolite concentrations for enzyme assay with NADP+-dependent glucose-6-phosphate dehydrogenase.

B, *In vivo* and *in vitro* metabolite concentrations for enzyme assay with phosphate acetyltransferase.

C, In vivo and in vitro metabolite concentrations for enzyme assay with fructose-bisphosphate aldolase class II.

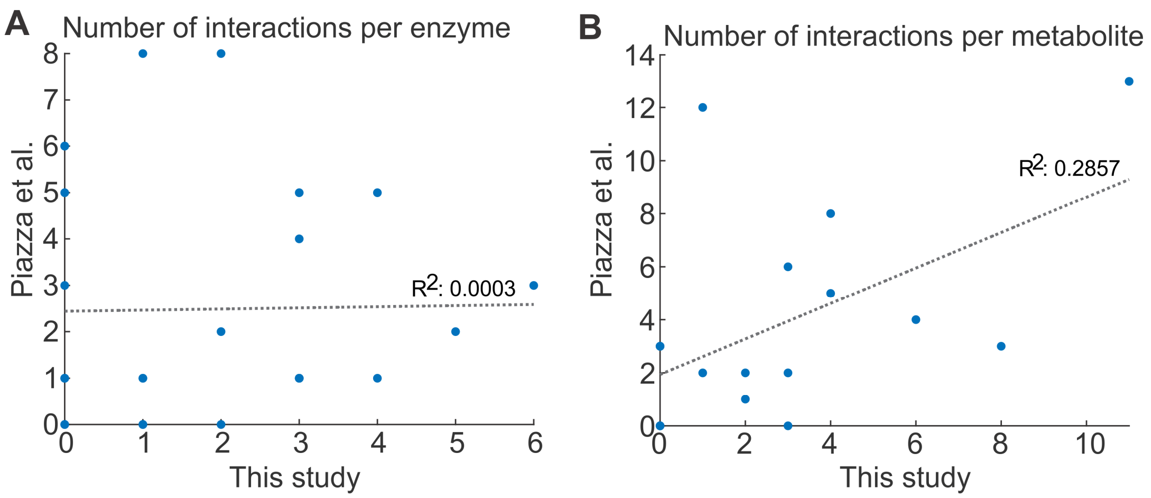


**Appendix Figure S11: Correlation of interactions detected per enzyme and metabolite in this study and Piazza et *al.* 2018.**

**A**, Correlation of the number of interactions detected per protein. The grey dotted line represents a linear fit with $R^2 = 0.0003$.

**B**, Correlation of the number of interactions detected per metabolite. The grey dotted line represents a linear fit with $R^2 = 0.2857$.

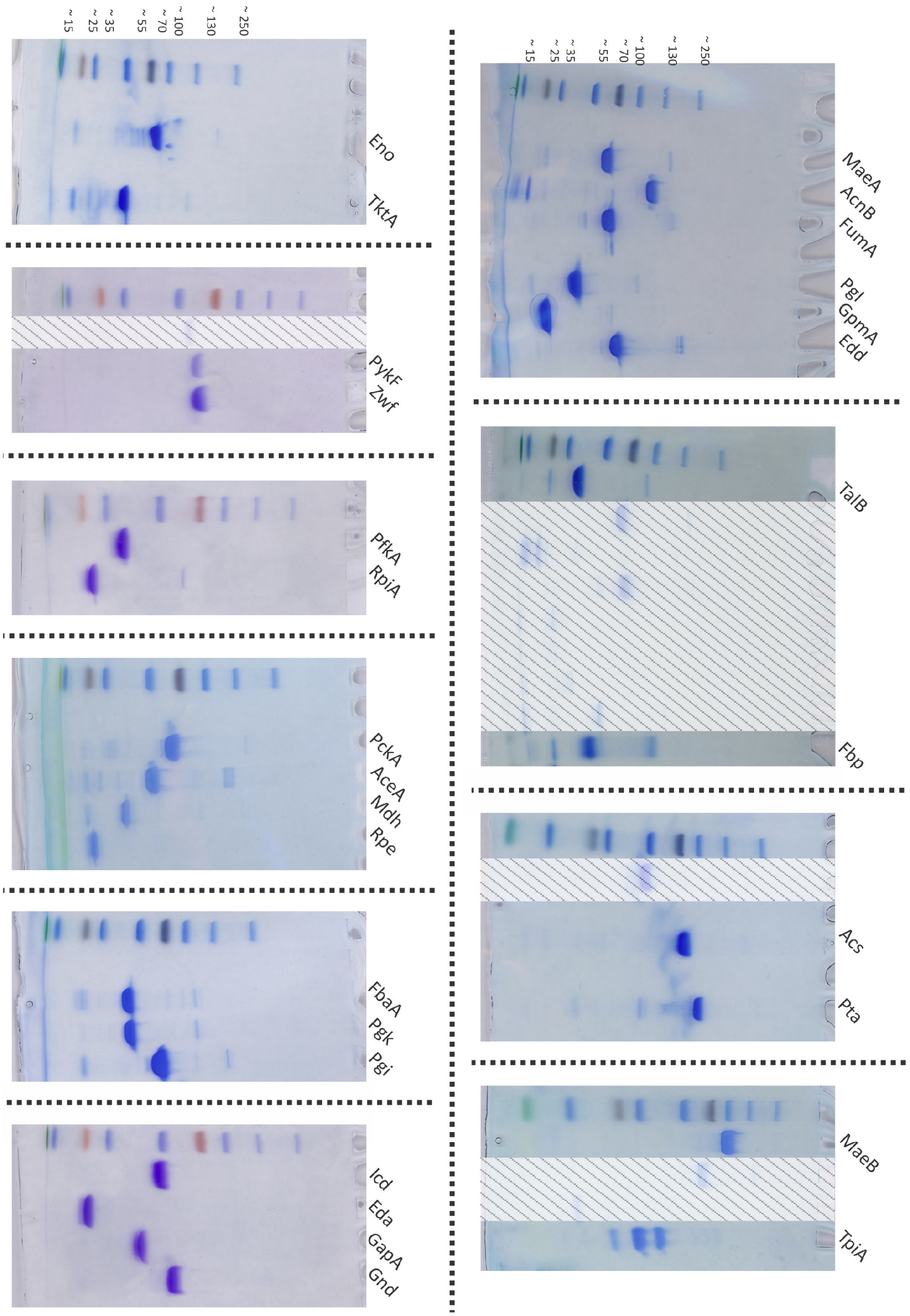


**Appendix Figure S12: SDS-PAGE of all tested proteins.**

The approximate marker sizes are indicated in kDa (Precision Plus Protein Dual Color standard, Bio-Rad).

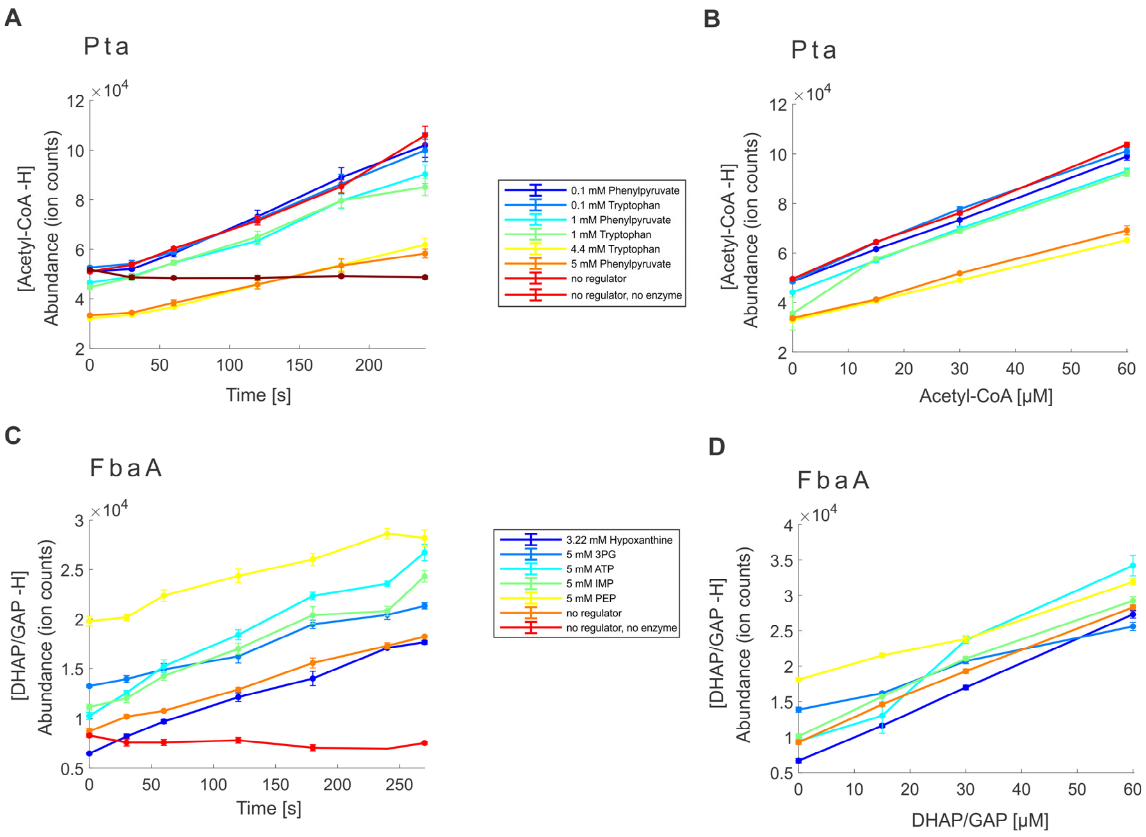


**Appendix Figure S13: Raw data for enzyme assays with Pta and FbaA.**

**A**, Increase of acetyl-CoA (product of Pta) over time, n= 3.

**B**, Standard curve for acetyl-CoA in the presence of different amounts of inhibitors (without added enzyme).

**C**, Increase of dihydroxyacetone phosphate/ glyceraldehyde-3-phosphate (products of FbaA) over time, n= 3.

**D**, Standard curve for dihydroxyacetone phosphate/ glyceraldehyde-3-phosphate in the presence of different amounts of inhibitors (without added enzyme).
